# Whitefly-tolerant transgenic common bean (*Phaseolus vulgaris*) line

**DOI:** 10.3389/fpls.2022.984804

**Published:** 2022-08-25

**Authors:** Amanda Lopes Ferreira, Josias Correa de Faria, Matheus da Costa Moura, Antônia Lopes de Mendonça Zaidem, Carolina Senhorinho Ramalho Pizetta, Elínea de Oliveira Freitas, Gesimária Ribeiro Costa Coelho, Jose Francisco Arruda e Silva, José Alexandre Freitas Barrigossi, Lucia Vieira Hoffmann, Thiago Lívio Pessoa Oliveira de Souza, Francisco José Lima Aragão, Patricia Valle Pinheiro

**Affiliations:** ^1^Laboratory of Entomology, Embrapa Arroz e Feijão, Santo Antônio de Goiás, Brazil; ^2^Laboratory of Biotechnology, Embrapa Arroz e Feijão, Santo Antônio de Goiás, Brazil; ^3^Escola de Agronomia, Universidade Federal de Goiás, Goiânia, Brazil; ^4^Laboratory of Genetic Engineering Applied to Tropical Agriculture, Embrapa Recursos Genéticos e Biotecnologia, Brasília, Brazil; ^5^Laboratory of Biotechnology, Embrapa Algodão, Santo Antônio de Goiás, Brazil

**Keywords:** dry bean, *Bemisia tabaci*, RNA interference, insect pest management, *vATPase*

## Abstract

Common bean (*Phaseolus vulgaris* L.) is a staple food in Brazil with both nutritional and socioeconomic importance. As an orphan crop, it has not received as much research attention as the commodity crops. Crop losses are strongly related to virus diseases transmitted by the whitefly *Bemisia tabaci*, one of the most important agricultural pests in the world. The main method of managing whitefly-transmitted viruses has been the application of insecticides to reduce vector populations. Compared to chemical vector control, a more sustainable strategy for managing insect-borne viruses is the development of resistant/tolerant cultivars. RNA interference has been applied to develop plant lines resistant to the whitefly in other species, such as tomato, lettuce and tobacco. Still, no whitefly-resistant plant has been made commercially available to date. Common bean is a recalcitrant species to *in vitro* regeneration; therefore, stable genetic transformation of this plant has been achieved only at low frequencies (<1%) using particle bombardment. In the present work, two transgenic common bean lines were obtained with an intron-hairpin construct to induce post-transcriptional gene silencing against the *B. tabaci vATPase* (Bt-*vATPase*) gene, with stable expression of siRNA. Northern blot analysis revealed the presence of bands of expected size for siRNA in leaf samples of the line Bt-22.5, while in the other line (11.5), the amount of siRNA produced was significantly smaller. Bioassays were conducted with both lines, but only the line Bt-22.5 was associated with significant mortality of adult insects (97% when insects were fed on detached leaves and 59% on the whole plant). The expression of the Bt-*vATPase* gene was 50% lower (*p* < 0.05) in insects that fed on the transgenic line Bt-22.5, when compared to non-transgenic controls. The transgenic line did not affect the virus transmission ability of the insects. Moreover, no effect was observed on the reproduction of non-target organisms, such as the black aphid *Aphis craccivora*, the leafminer *Liriomyza* sp. and the whitefly parasitoid *Encarsia formosa*. The results presented here serve as a basis for the development of whitefly-tolerant transgenic elite common bean cultivars, with potential to contribute to the management of the whitefly and virus diseases.

## Introduction

The whitefly *Bemisia tabaci* (Genn.; Hemiptera: Aleyrodidae) biotype MEAM1 is currently considered one of the most important crop pests worldwide, for several reasons, including its wide geographic distribution, in all continents, strong performance as a vector of plant viruses and ability to colonize several plant families. Moreover, this insect presents high adaptability to different environments and rapid selection of insecticide-resistant populations. Whiteflies are a threat to food security, especially for developing countries (De Barro et al., 2011). As a generalist insect, *B. tabaci* feeds on a wide range of host plants, including common beans, cotton, tomatoes and soybeans. For those reasons, in countries with a tropical climate, *B. tabaci* can be found in both cultivated areas and native vegetation throughout the year, placing this insect among the ten most invasive pests in the world (Chen et al., 2016). In addition to the direct constraint caused by feeding on the plant, the whitefly is responsible for the transmission of several plant viruses, which is considered the main damage associated with this insect in agricultural crops. *B. tabaci* is the exclusive vector of viruses from the genus Begomovirus.

Common bean (*Phaseolus vulgaris* L.) is a staple food in Brazil with nutritional and food security importance, as a relevant source of protein. It is also a crop of substantial impact on the Brazilian agribusiness, because it is produced in all regions of the country, in three cropping seasons per year, with a diversified use of technology. The majority of the common bean production in Brazil is carried out in small-holder farmers, providing employment and income to family producers. On the other hand, the crop is also produced by industrial farmers, in larger areas, with supplementary irrigation mainly in the Central Brazil growing area.

One of the main challenges of the crop is the high incidence of virus diseases, the most important ones transmitted by *B. tabaci*. Losses of up to 100% have been reported due to damages associated with the Begomovirus bean golden mosaic virus (BGMV; Souza et al., 2016). In addition to this virus, the whitefly transmits other viruses to common beans and soybeans, such as the Carlavirus cowpea mild mottle virus (CPMMV) and a recently reported Cytorhabdovirus (Alves-Freitas et al., 2019; Pinheiro-Lima et al., 2020). Currently, the main method of managing whitefly-transmitted viruses has been the intensive use of insecticides to reduce the vector population. However, the intense use of the same insecticide molecules, often not associated with other management techniques, has rapidly reduced the efficacy of insecticides and selected whitefly populations resistant to the majority of the active ingredients on the market, thus limiting the efficiency of chemical control. In recent years, there are no records of new insecticides to control this pest, which indicates a limitation in the development of new synthetic molecules. Furthermore, the excessive use of synthetic pesticides poses a risk to human health and to the environment, in addition to increasing production costs. It is not difficult to find reports of 20 applications per common bean crop season for the management of this insect pest (Souza et al., 2016).

A more sustainable strategy for pest management is the development of pest resistant/tolerant plant cultivars. Strategies for the development of commercial cultivars resistant/tolerant to whitefly-transmitted viruses have been developed, for example, the transgenic common bean cultivar BRS FC401 RMD, which is resistant to BGMV (Bonfim et al., 2007; Faria et al., 2016; Souza et al., 2016) and the tomato cultivar BRS Sena, tolerant to Bemogoviruses (Quezado-Duval et al., 2014). However, considering the plasticity of the “virus transmission ability” phenotype of *B. tabaci*, as well as its high adaptation to a wide range of environments and hosts, plant breeding for resistance to plant virus may contribute to the virus disease management, but not to the management of other viruses transmitted by this insect vector. As an efficient vector of plant viruses, even a single adult whitefly is capable of carrying and transmitting different species of viruses, acquired from mixed-infected plants.

Although there is no commercially available whitefly-resistant plant line yet, some reports show the development of whitefly-resistant plants by stable genetic transformation, such as tomato, tobacco and lettuce (Ibrahim et al., 2017; Pizetta et al., 2021; Xia et al., 2021), but not common beans. Our team has developed the first transgenic common bean cultivar in the world, resistant to BGMV, which has recently been made commercially available. Although some common bean cultivars have been reported to present tolerance to the whitefly through antixenosis (Silva et al., 2014, 2019; Hoshino et al., 2017; Jesus et al., 2021), using interfering RNA (RNAi) to silence important genes in the insect is also a promising strategy, because it can be more specific to the target insect and generally leads to high mortality. Silencing the insect *vATPase* gene (Bt-*vATPase*), using RNAi in Hemipteran insects has proven to reduce survival and to interfere in the development of juvenile stages, including *B. tabaci* (Thakur et al., 2014; Ibrahim et al., 2017). The ATPase enzyme is part of the family of ATP-dependent proton pumps located in a variety of eukaryotic cell membranes. It is responsible for controlling pH in intracellular compartments and its activity affects several cellular processes, such as intracellular membrane transport, processing and transport of neurotransmitters, as well as regulating the entry of viruses and microorganisms. Here we report the development of the first transgenic common bean line with tolerance to the whitefly *B. tabaci*, by silencing the insect *vATPase* gene, using RNAi. We generated two transgenic lines and one of them was tolerant to the whitefly, causing significant mortality of adult insects. The next step will be to transfer the transgene to elite common bean lines for possible commercial use by farmers after biosafety studies.

## Materials and methods

### Insect colonies

The whiteflies *Bemisia tabaci* MEAM1 biotype used in the experiments were originated from a colony on common bean (*P. vulgaris*, cv. Pérola), kept under screenhouse conditions, at Embrapa Arroz e Feijão, Santo Antônio de Goiás, GO, Brazil (16° 28′ 00” S, 49° 17′ 00” W; 823 m asl), as previously described (Pizetta et al., 2021). To obtain age-synchronized adult insects, plants containing whitefly eggs laid for 2 h were isolated in insect cages, after removing the adults, until reaching the fourth larval instar. Adult insects used in the mortality experiments were collected 1 day after the onset of adult emergence.

A colony of the black aphid *Aphis craccivora* was obtained from bean plants collected at Embrapa Arroz e Feijão and maintained on common bean plants isolated in insect cages. The whitefly parasitoid *Encarsia formosa* was obtained from a colony maintained on whitefly nymphs fed on kale (*Brassica oleracea*) plants.

### Genetic transformation

A partial sequence of 647 bp from the *B. tabaci v-ATPase* gene was cloned in sense and antisense orientations in the vector pSIU (Tinoco et al., 2010) generating pBtATPase, as previously described (Ibrahim et al., 2017), for genetic transformation of the common bean (Supplementary Figure S1). The ATPase interference cassette is under the control of the doubled 35SCaMV promoter with an enhancer sequence from the alfalfa mosaic virus (dCaMV35S) and the terminator is that of the *nopaline synthase* gene (*nos*). The selection gene used was the *Atahas*, with the complete promoter and terminator from *Arabidopsis thaliana*, conferring tolerance to the herbicide imazapyr. The RNAi construct will be referred to as *ΔATPase* from now on.

Genetic transformation of the common bean cultivar Olathe Pinto was performed as described (Aragão et al., 1996; Bonfim et al., 2007). Briefly, common bean seeds were surface disinfested in 70% ethanol (V/V) for 1 min, followed by immersion in 2.5% sodium hypochlorite for 10 min. Soon after, three washes were performed using sterile water. After the last wash, the seeds were soaked in sterile water for approximately 18 h. After this period, the seed embryonic axis was excised and their apical meristems exposed after the removal of the primordia of the primary leaves (plumule), with the aid of a stereoscopic microscope. Then, they were placed in sterile Petri dishes (60x15mm) containing MS medium amended with phytagel, with the apical meristem facing the center of the dish. Particle bombardment of DNA was performed using a particle accelerator as described by Sanford (1990), Klein et al. (1992). Embryos were transferred to plant tissue culture containers with selective culture medium containing 6-benzylaminopurine (BAP; 10 mg/l) and imazapyr (80 nm), which were kept in a growth chamber at 24°C and 16 h photoperiod. The explants that developed and were positive for the presence of *Atahas* gene by PCR were transferred to a container with sterile substrate, covered by a plastic bag that was gradually removed so that the explants could acclimate to the environment. After this process, they were transferred to pots with soil and fertilizer and kept in a greenhouse to complete the development and for PCR analysis. For that, DNA was isolated from leaf tissues as described (Dellaporta et al., 1983) and amplified by PCR with the following pair of primers: AHASP124F 5′ACTAGAGATTCCAGCGTCAC3´ and AHAS500CR 5′GTGGCTATACAGATACCTGG3´ for the detection of the selection gene *Atahas.* Thermal cycling conditions were denaturation at 95°C for 15 min followed by 35 cycles of 94°C for 1 min, 56°C for 1 min, 72°C for 1 min, and 60°C for 30 min.

### Progeny analysis

Segregation ratio was evaluated at the second and third generations (T_2_ and T_3_) of self-pollinated transformed plants, analyzing the presence of the *ΔATPase* by PCR, as described. Pearson’s Chi squared (*χ*^2^) was used to determine whether the observed segregation ratio was consistent with a Mendelian ratio of 3:1, at 95% level of confidence. Homozygous plants were used for the reported bioassays.

### Production of *ΔATPase* siRNAs

Leaf samples from 10-day-old plants were collected in liquid nitrogen for total RNA isolation, using Trizol (Invitrogen), as recommended by the manufacturer. Non-transgenic plants with the same genetic background (cv. Olathe pinto) and the same age were used as controls. SiRNA analysis was performed as described (Bonfim et al., 2007; Pizetta et al., 2021), using a DNA probe corresponding to the *vATPase* PCR fragment, which was amplified using the primer pair ATPXS1 (TTCTAGAGCTCTATCACACTATCTGAGTAC)/ATPSK1(GGTACCACTAGTGGGAAGTTTTTATCGTAG) labeled with α^32^P dCTP and the DecaLabel DNA Labeling Kit (Thermo Fisher Scientific), according to the manufacturer’s instructions. The bands were visualized with a fluorescent image analyzer (FLA-3000; Fujifilm).

### Whitefly mortality assays

**Figure 1:**
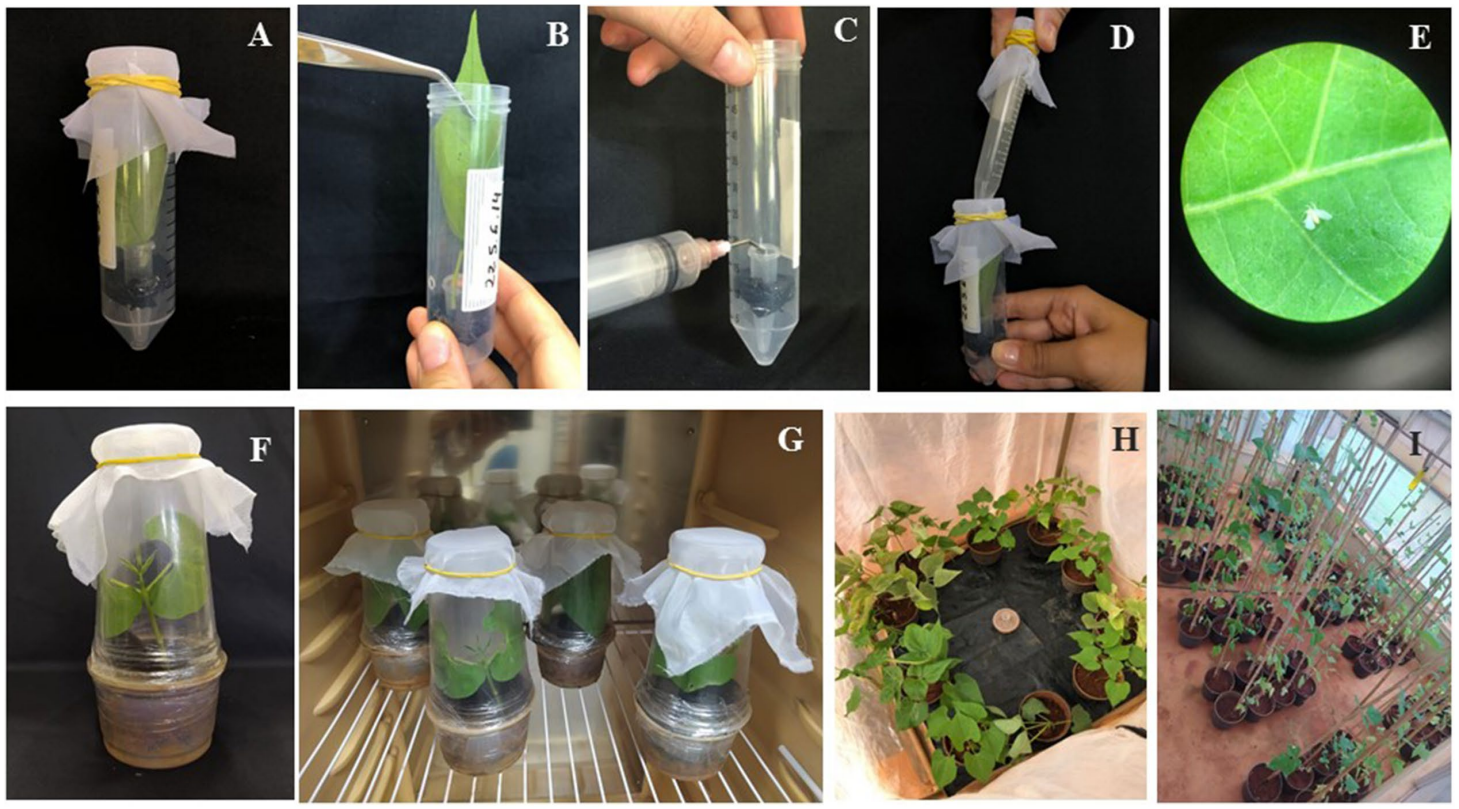
Whitefly mortality and preference assays. **(A)** Bioassay system developed to conduct the insect mortality assays with detached leaves of the transgenic common bean lines, with voile fabric covering the upper part of the tube; **(B)** Plant leaf being inserted into the bioassay system; **(C)** Microtube used to place water and maintain the leaf for the period of the evaluations; **(D)** Release of adult insects inside the Falcon tube; **(E)** Close-up photo of dead insect on GM bean leaf; **(F)** Plastic cup cage to isolate the plants; **(G)** Plants in the growth chamber for the whole-plant experiment; **(H)** Transgenic and non-transgenic common bean plants randomly distributed in a circle under a large voile cage, where insects were released in the center, for the preference assays and **(I)** Transgenic and non-transgenic common bean plants distributed in blocks in the greenhouse for the experiment during the common bean cycle.

Two sets of bioassays were conducted for each common bean transgenic line, the first one using detached leaves and the other with the whole plant. To keep the detached leaves during the experiments, a bioassay system was developed using 50 ml Falcon tubes, containing 1.5 ml microtubes fixed to the bottom (Figure 1A). The detached leaves were accommodated with the petioles inside the microtubes containing water (Figures 1B,C) and the system was covered with voile fabric. Each replicate consisted of one detached leaf from an individual transgenic or non-transgenic plant (*n* = 15) and 20 two-day-old adult insects carefully collected from the colony with the aid of 15 ml Falcon tubes with one end opened (Figure 1D). The evaluations were carried out 5 days after the assembly of the assay, counting the number of live and dead adults on the leaves (Figure 1E), with the aid of an insect aspirator. This bioassay was repeated twice for each transgenic line.

For the experiments with whole plants, transgenic and non-transgenic plants (*n* = 10) with the primary leaves fully expanded were submitted to whitefly oviposition for 1 h. After that, adult insects were removed from the leaves and plants were kept on a growth chamber (25°C and 16 h:8 h light/dark photoperiod) during the insect development from egg to adult, for about 20 days. When the insects reach the fourth instar, plants were isolated in individual cages, made of plastic cups covered with voile fabric, to avoid the scape of adult insects (Figures 1F,G). 7 days after adult emergence, the numbers of live and dead adults and empty pupae were counted on each plant.

### Gene silencing in insects

The expression levels of the *vATPase* gene in *B. tabaci* feeding on transgenic (T_3_ generation; line Bt-22.5) and non-transgenic common beans were determined by qRT-PCR. About 150 two-day-old adult insects were transferred to transgenic and non-transgenic plants (*n* = 3) isolated in individual cages. After 48 h, insects were collected using an insect aspirator coupled to microtubes, which were immediately placed on liquid nitrogen. Total RNA was isolated from ~150 adult insects/replicate, and 200 ng of RNA from each sample were used to synthesize the cDNA with the Promega GoScript Reverse Transcription System kit, according to manufacturer’s instructions. PCR reactions were performed using the Step OnePlus real-time PCR system (Thermo Fisher Scientific) with SYBRGreen detection. Primers for the *vATPase* and *actin* genes were designed using the PrimerQuest tool (IDT Integrated DNA Technologies, Inc.), as described (Pizetta et al., 2021). The relative levels of *vATPase* transcription in the different RNA samples were normalized in relation to the *actin* gene, an internal standard. Quantitative assays were performed using three biological samples. The relative level of expression was calculated using the Livak method (Livak and Schmittgen, 2001).

### Preference and oviposition assays

Pots with transgenic or non-transgenic plants (*n* = 5) were placed inside a large voile fabric cage (1.5 mL × 1.5 mW × 1.5 mH), in a circle, randomly distributed (Figure 1H). In the center of the cage, 400 adult whiteflies were released. The number of adult insects sitting on the primary leaves of the plants was counted 48 h later, using a small mirror to prevent the insects from leaving the plants due to the movement of the foliage. After counting the number of adults, one leave of each plant was removed and taken to the laboratory for egg counting under a stereoscopic microscope.

### Effect of the transgenic common bean line on virus transmission by the whitefly

To evaluate the potential effect of silencing the *vATPase* gene in the whitefly on virus transmission by the insect, we conducted transmission assays with two economic important viruses of common beans: cowpea mild mottle virus (CPMMV), which has a mild effect on common beans, and bean golden mosaic virus (BGMV), which cause a severe mosaic and stunting on susceptible plants. Common bean plants cv. BRS Pérola, susceptible to both viruses, were used as the inoculum source. After an acquisition period of 24 h, viruliferous whiteflies were transferred to two individual cages, one of them containing a common bean plant from line Bt-22.5 and the other one containing a non-transgenic common bean Olathe plant. Insects were kept on these two treatments for 48 h. After that, 100 insects were transferred from each treatment to larger cages, containing 30 recipient plants (Olathe Pinto, non-transgenic) for a 24 h inoculation period. Then, all adult insects were manually removed from the plants, using an insect aspirator, and plants which were kept in cages for 25 days, for virus infection evaluation. Virus incidence was assessed by visual symptoms, using a 1–4 scoring scale, in which 1 = no symptom, 2 = light symptoms, 3 = moderate symptoms and 4 = strong symptoms (Arias et al., 2015). Virus detection was analyzed on symptomatic and asymptomatic plants (*n* = 12) by PCR and RT-PCR, using the primers CPMMV-F 5′ACGTCTCGAGCTGGAGTCAGTGTTTG3′/CPMMV-R (5′ACGTGAATTCTTACTTCTTAGCGTG3′) and BGMV_pAC1v1978 (5′GCATCTGCAGGC CCACATYGTCTTYCCNGT 3′) / BGMV_ pAV1c715 (5′GATTTCTGCAGTTDATRTTYTCRT CCATCCA 3′).

### Effect of the transgenic common bean line on two whitefly generations

A greenhouse experiment was carried out to simulate the effect of the transgenic line Bt-22.5 on the whitefly population in the field, because the field release has yet to be requested to the Brazilian National Biosafety Technical Commission (CTNBio). The experiment was carried out in a randomized block design. Transgenic and non-transgenic seeds, 48 of each, were sown in large plant pots and randomly distributed in six blocks inside the greenhouse, to mitigate the potential effect of spots with different light incidence. Each block was composed by 8 plants of each treatment (Figure 1I). When the plants had the two primary leaves fully expanded, kale plants hosting fourth instar nymphs of the whitefly were randomly distributed among the blocks, so that the emerging adults could freely move to the common bean plants. The whitefly-source kale plants were kept in the greenhouse for 2 days and then they were removed. The number of adult whiteflies sitting on the plants were sampled in 18 plants/treatment weekly. From the third week on, leaves from 18 different plants/treatment were randomly collected every week and analyzed in the lab, using a stereoscope microscope, to count the number of eggs, nymphs and empty pupae. Data were collected weekly, until the plants enter the R8 stage (pod filling), comprising 2.5 whitefly generations. Pods from three plants/treatment/block were harvested at the end of the plant cycle to evaluate the number of pods per plant, seeds per pod and mass of 100 seeds. Seed mass was corrected at 13% moisture. The mass of 100 seeds was estimated from the mass of seeds collected from three plants.

### Bioassays with non-target organisms

To evaluate the potential effect of the transgenic common bean line Bt-22.5 on a non-target organism that feeds directly on bean leaves, with a feeding habit similar to the whitefly, we evaluated the reproduction of the black aphid *A. craccivora*. Five 4th instar nymphs of *A. craccivora* were carefully transferred to each primary leaf of transgenic (Bt-22.5) and non-transgenic (cv. Olathe Pinto) plants (*n* = 4), using a soft wet paintbrush. Leaves were isolated with individual little bags, made of voile fabric. Plants were then kept on a growth chamber at 25°C and 16 h light: 8 h dark photoperiod. 7 days later, the total number of aphids in each plant was counted using a stereoscope microscope. In another set of experiments, the whitefly parasitoid *E. formosa* was used to evaluate a potential indirect effect of the transgenic common bean line to a beneficial non-target organism. Transgenic and non-transgenic common bean plants (*n* = 10, considering each primary leaf as a replicate), with their primary leaves fully expanded, were submitted to whitefly oviposition for 2 h. After that, the adults were removed and plants were isolated in cages. The apical leaves were pruned to avoid excessive plant growth. When the nymphs reached the 3rd instar, the plants were randomly distributed in a circle, in the middle of which a kale (*Brassica oleracea*) plant containing adults of *E. formosa* was placed, so that the parasitoids could move to the common beans to parasitize the whitefly nymphs. 2 days later, the adult parasitoids were manually removed from the common bean plants, using an insect aspirator. After 12 days, close to the parasitoid emergence, common bean leaves were collected to sample the number of parasitized nymphs and non-parasitized nymphs. These experiments were repeated twice and data from both experiments were analyzed together.

Additionally, during the greenhouse experiment to look at the effect of the transgenic line Bt-22.5 on the whitefly over two generations, a natural infestation of the leafminer *Liriomyza* sp. occurred, severely damaging the plants, because the common bean cultivar Olathe Pinto is highly susceptible to this insect pest and it was not possible to use insecticides without affecting the whitefly population. Then we included an assessment of the occurrence of the *Liriomyza* sp. larvae on the transgenic and non-transgenic plants, as another non-target insect species. The number of larvae was counted in three leaves per plant, and 3 plants/block (*n* = 18). The level of damage on the leaves was evaluated in the same leaves (*n* = 18), using a scoring scale from 1 to 4 (1 = no mining; 2 = a few mines in less than 20% of the leaflets, no defoliation; 3 = mines present in up to 50% of the leaflets, some defoliation leaflets; 4 = many mines in almost all the leaflets (90%) and defoliation of greater than 31%.), adapted from (Singh and Weigand, 1994).

### Statistical analysis of the bioassays data

The homogeneity of variances was verified by the Levene test and data normality by the Shapiro–Wilk test. Means of normally distributed data were compared using the t test (*p* < 0.05). Non-parametric data was analyzed using the Wilcoxon test. In the preference assay, the number of eggs and adults was modeled using the GLM with Binomial Negative distribution. In the experiment to look at the whitefly generations on the common bean plants, the total number of adult insects, eggs, empty pupae and nymphs per treatment were analyzed using the above mentioned tests, considering block effects. For the scoring scales, the analysis was performed considering the frequency of each of the scoring scale per treatment. These frequencies were compared by the chi-square value and by a proportion test, where the null hypothesis indicated that the percentage of plants with a certain score was similar in the two treatments. All statistical analysis were performed using the R software (R CORE TEAM, 2019).

## Results

### Analysis of common bean transgenic plants

**Figure 2:**
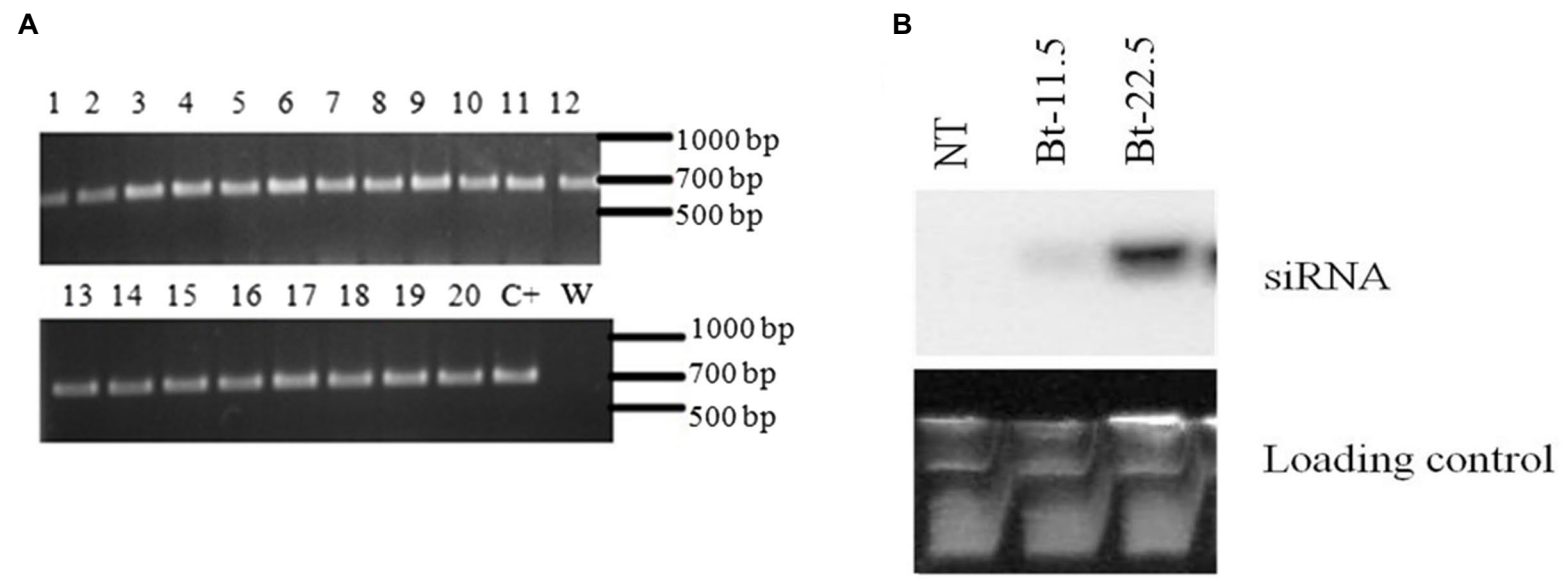
Analysis of common bean transgenic plants and relative expression of the *vATPase* gene in the whitefly *Bemisia tabaci*. **(A)** Progeny analysis of the transgenic common bean line Bt-22.5 for the marker gene *ahas*. Numbers 1 to 20 correspond to the 20 T_2_ plants from seeds collected from plant Bt-22.5, C+ is the positive control and W is water, used as negative control. **(B)** Northern blot analysis for the detection of *Bemisia tabaci* vATPase small interfering RNA (siRNA) isolated from transgenic common bean lines Bt-11.5 and Bt-22.5. NT is the non-transgenic common bean cv. Olathe Pinto. SYBR Safe stained RNA served as loading control.

In 44 transformation attempts, 8,764 explants were subjected to particle bombardment for genetic transformation. Of these, only nine T_0_ plants were positive for the presence of the *ΔvATPase* transgene, resulting in a low rate of transformed plants (0.1%), as expected (Russell et al., 1993; Aragão et al., 1996). From the nine T_0_ plants, only two transmitted the transgene to the progeny (T_1_). These two lines were named 11.5 and Bt-22.5. Among the 9 T_1_ plants of the line 11.5 obtained, 7 plants were positive for the transgene, while for the line Bt-22.5, 3 of the 7 plants were positive. Seeds collected from individual self-pollinated T_1_ plants were sowed for the progeny analysis of the T_2_ (line Bt-22.5) and T_3_ (line 11.5) generations (*n* = 20). Most of these lines did not segregate as expected (Table 1). However, all 20 plants from the progeny of the line Bt-22.5 were positive for the selection gene *Atahas*, indicating that this line was homozygous for the transgene *ΔATPase* (Table 1; Figure 2A). A similar pattern was observed for line Bt-22.5.6, in which, 19 of the 20 plants were positive for the transgene (Table 1).

**Table 1:** Progeny analysis of T_2_ and T_3_ generations of transgenic common bean cv. Olathe Pinto lines (*n* = 20).

Common bean line	Generation	Positive^a^	Negative^a^	*χ* ^2^	*P* ^b^
11.5.1.3	T3	12	8	1.7	0.200
11.5.2.4	T3	8	12	11.3	0.001
11.5.3.5	T3	10	10	5.4	0.020
11.5.4.19	T3	9	11	8.1	0.005
11.5.5.12	T3	15	5	0.0	1.000
11.5.6.21	T3	10	10	5.4	0.020
11.5.7.23	T3	7	13	15.0	0.0001
Bt-22.5.6	T2	19	1	3.3	0.07
Bt-22.5.5	T2	20	0	5.4	0.020
Bt-22.5.2	T2	10	10	5.4	0.020

a Data are based on PCR analysis for detection of the ΔATPase transgene.

b Probability of the observed segregation fits the expected 3:1 Mendelian ratio at 95% confidence interval.

Northern blot analysis revealed that plants from lines 11.5 and Bt-22.5 produced siRNA bands corresponding to the expected size range (Figure 2B). However, the siRNA band from line 11.5 was weaker than that of line Bt-22.5. No signal was observed for the non-transgenic control plants.

For the transgenic line Bt-22.5, no phenotypical difference was observed, compared to the non-transgenic plants. Additionally, the number of pods per plant, seeds per pod and the mass of 100 seeds did not present significant difference between treatments (Supplementary Table S1).

### Effect of the transgenic plants on the whitefly

**Figure 3:**
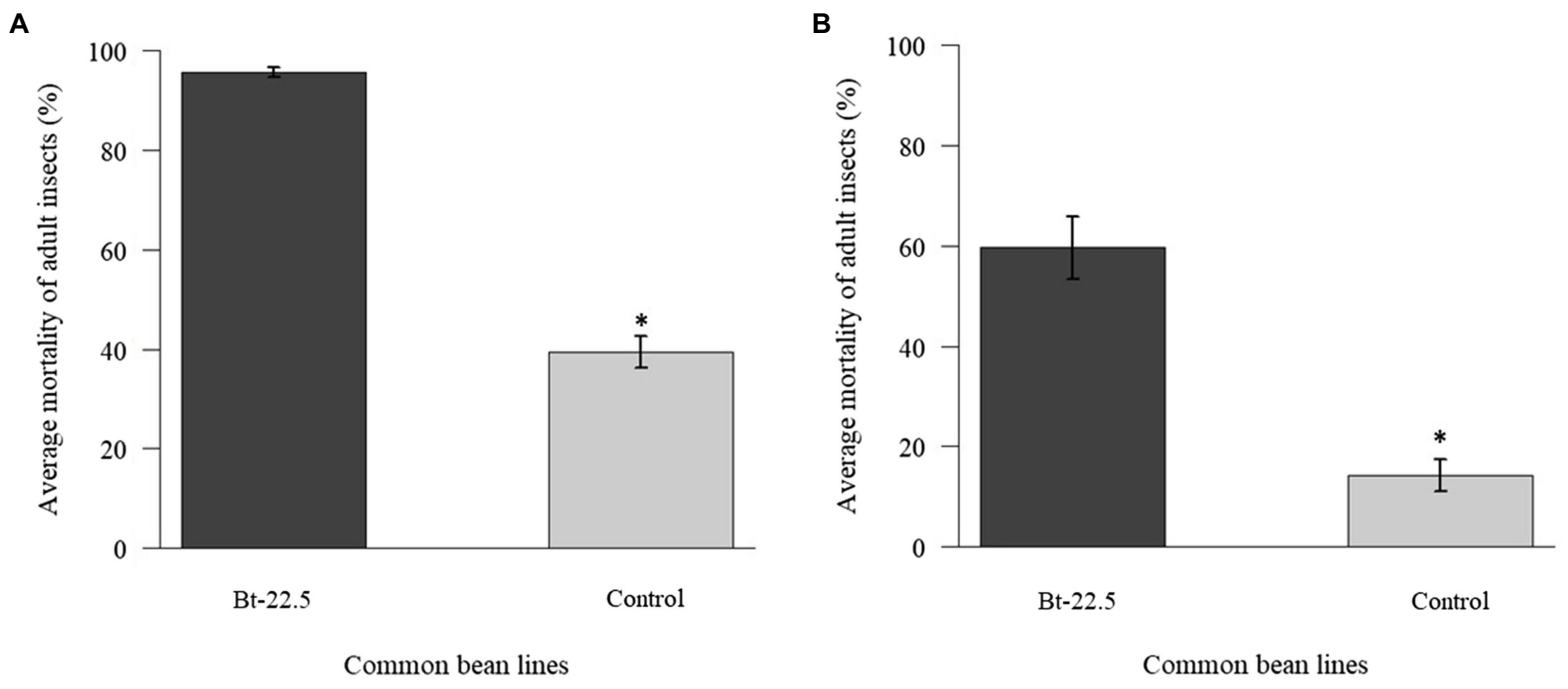
Mortality of adult whiteflies *Bemisia tabaci* after silencing their *vATPase* gene on the transgenic common bean line Bt-22.5. **(A)** Detached leaves (*n* = 15) and **(B)** whole plant (*n* = 10) experiments (^*^p < 0.05).

Mortality of adult whiteflies was significantly higher in the transgenic common bean line Bt-22.5 both for the detached leaf and for the whole plant experiments (Figure 3), compared to the controls. The experiment with detached leaves resulted in a higher mortality (97%) than the experiments with the whole plant assays (59%; Figure 3). In contrast, when insects fed on detached leaves of the line 11.5, mortality was not different from that observed in the control plants (data not shown).

**Figure 4:**
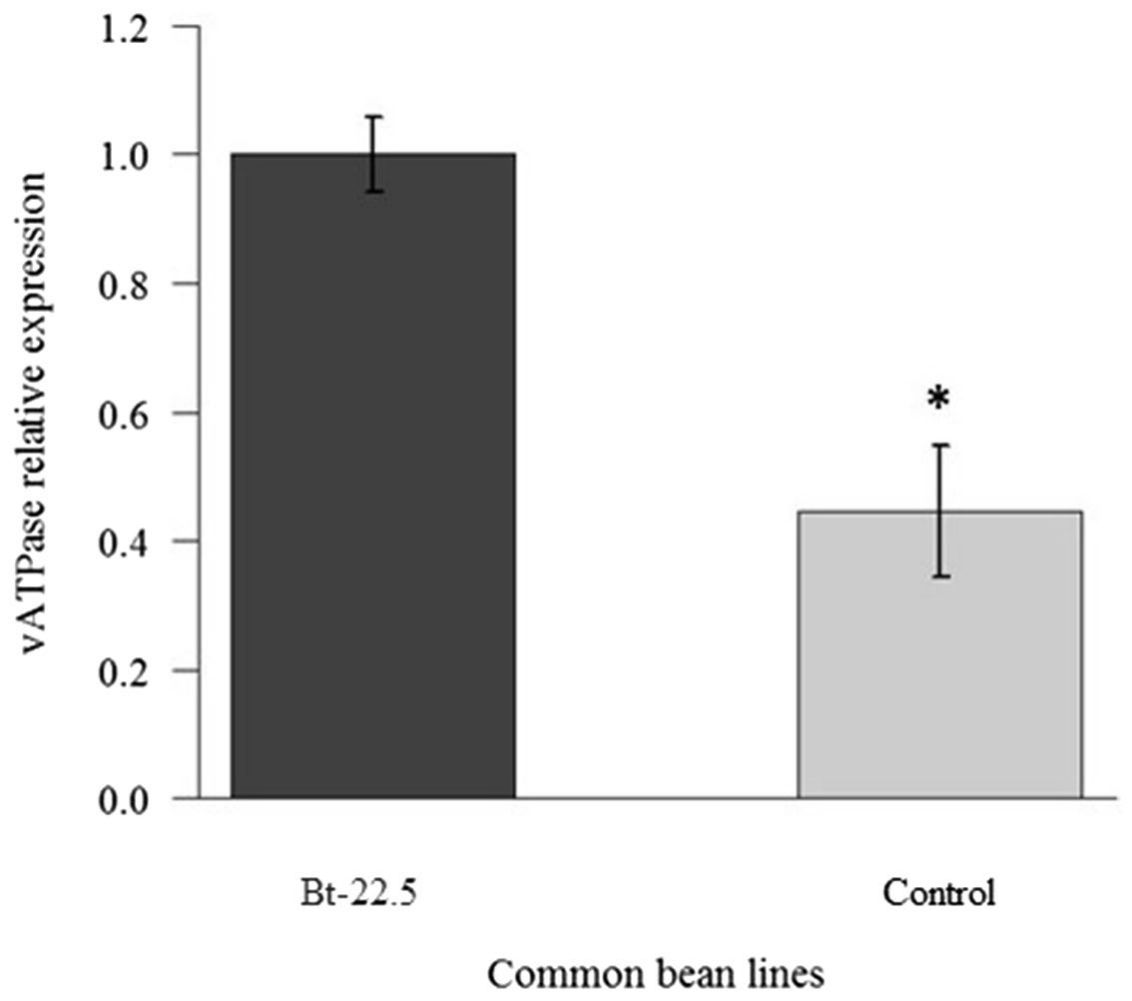
Relative expression of the transcripts of the *vATPase* gene in *Bemisia tabaci adults* after 48 h of feeding on the transgenic common bean plants, determined by qRT-PCR (^*^p < 0.05, *n* = 3).

**Figure 5:**
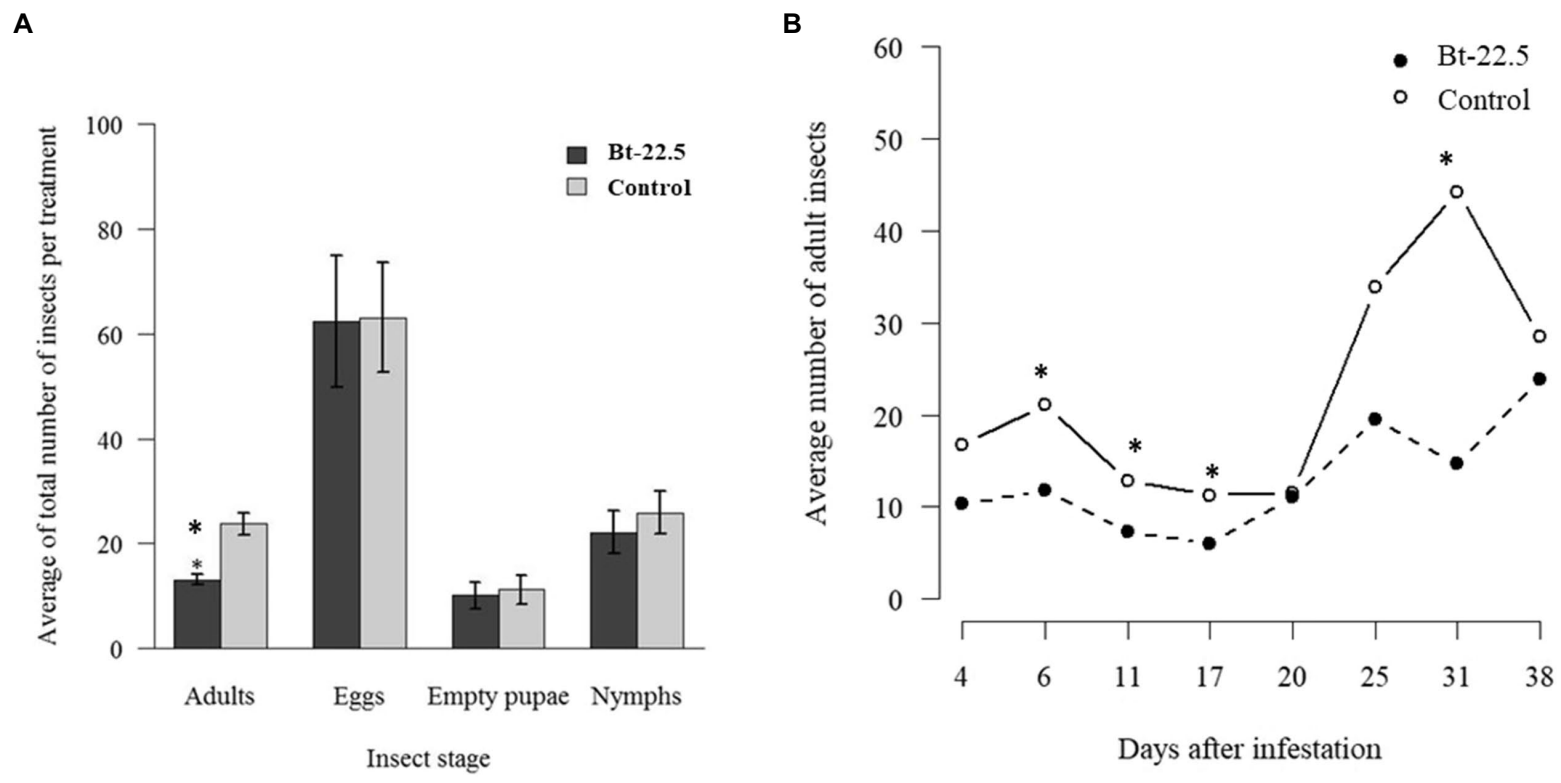
Development of the whitefly population during the cycle of the transgenic common bean plants. **(A)** Average of the total number of adult insects, eggs, empty pupae and nymphs per treatment; **(B)** Average number of adult insects per treatment in each sampling date (^*^p < 0.05).

Expression of the *vATPase* gene in insects was significantly reduced when they fed on the transgenic common bean line Bt-22.5, less than half of the expression observed in insects that fed on the control plants (Figure 4). In the preference assay, a reduced proportion of adult whiteflies (27.6%) and eggs (25.9%) was observed, on average, on the transgenic plants compared to the number of insects sampled on the control plants (data not shown). In the experiment to look at the whitefly generations during the cycle of the common bean plants, 2.5 whitefly generations were evaluated. The total number of eggs, empty pupae and nymphs did not differ between transgenic and non-transgenic plants (Figure 5A). However, the total number of adult insects was significantly lower on the transgenic plants (Figure 5A). Accordingly, the average number of adults per treatment was significantly higher on the control plants in four of the eight sampling dates (Figure 5B). Remarkably, when the second-generation adults began to emerge (May 3rd and May 9th), the population increased significantly faster in the non-transgenic controls, until the number of adults almost coincided in the last sampling date.

### Effect of the transgenic common bean line Bt-22.5 on virus transmission by the whitefly

**Figure 6:**
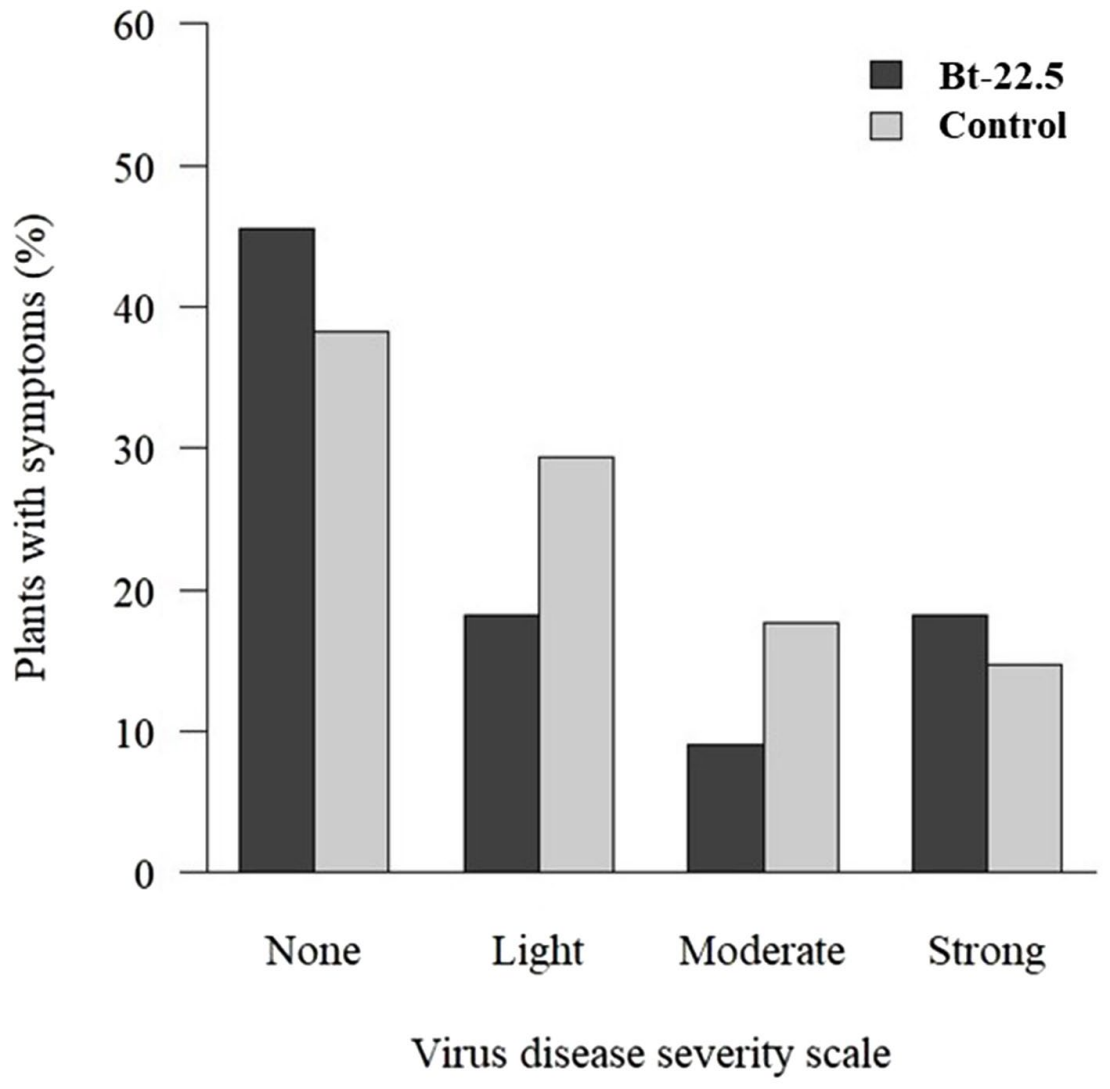
Proportion of transgenic and non-transgenic common bean plants with visual symptoms of virus disease, according to a scoring scale from 1–4, in which 1 = no symptom, 2 = light symptoms, 3 = moderate symptoms and 4 = strong symptoms (Arias et al., 2015).

Visual symptoms of virus disease were observed in 61.2% of the non-transgenic plants, while in the transgenic plants only 45.5% of the plants virus symptoms (non-significant; Table 2). For the plants that presented virus symptoms, the proportion of plants in each virus disease severity score was not different between treatments (Figure 6). Although many plants were asymptomatic, PCR analysis showed that the proportion of plants infected with CPMMV and BGMV did not differ between treatments (transgenic vs. non-transgenic; Table 2).

**Table 2:** Proportion of common bean cv. Olathe Pinto plants (non-transgenic) with virus symptoms, positive for BGMV and CPMMV by PCR, after inoculation by viruliferous whiteflies previously fed on the transgenic common bean line Bt-22.5 or on the non-transgenic plants for 48 h.

Virus detection	Transgenic line Bt-22.5	Non-transgenic line	Value of p
Plants with virus symptoms	10/22 (45.5%)	21/34 (61.2%)	0.355544
BGMV^+^ plants	2/12 (16.7%)	3/12 (25%)	0.932414
CPMMV^+^ plants	12/12 (100%)	12/12 (100%)	NA

### Non-target organisms

**Figure 7:**
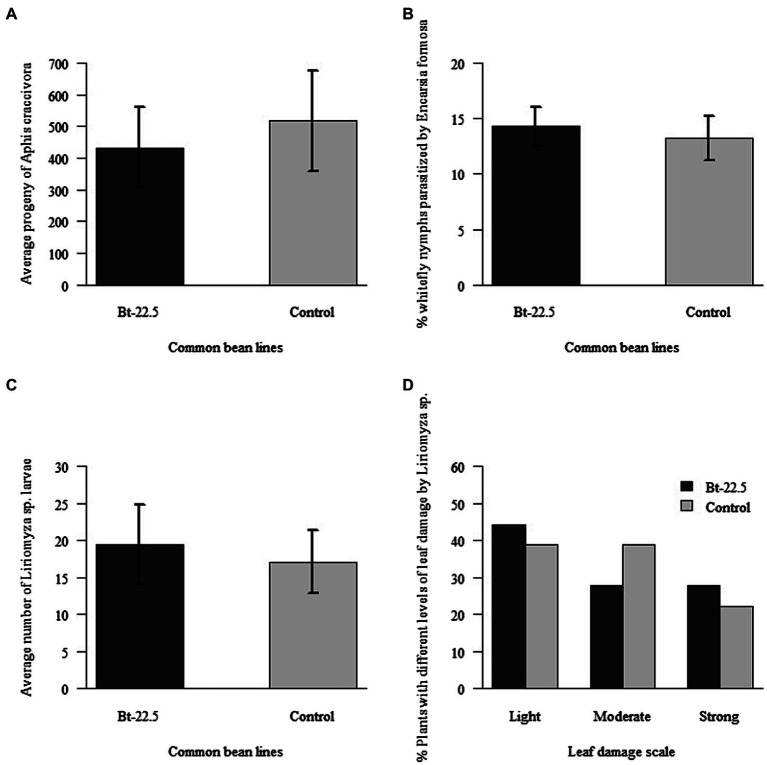
Development of non-target insects on plants of the transgenic common bean line Bt-22.5 expressing an RNAi construct for the silencing of the whitefly *Bemisia tabaci vATPase* gene, compared to the non-transgenic control: **(A)** Average progeny of the black aphid *Aphis craccivora* (*n* = 3); **(B)** Average percent of whitefly nymphs parasitized by *Encarsia formosa* (*n* = 3). **(C)** Average number of larvae of the leafminer *Liriomyza* sp. (*n* = 18); **(D)** Percent of plants with different levels of leaf damage from *Liriomyza* sp.

The reproduction of the black aphid *A. craccivora*, a non-target insect pest, was unaffected after feeding on the common bean transgenic line Bt-22.5 for 7 days (*p* < 0.05; Figure 7A). In the bioassays with the whitefly nymph parasitoid *E. formosa*, the number of parasitized whitefly nymphs in the transgenic plants was not significantly different from that of the non-transgenic plants (Figure 7B). Additionally, the average number of *Liriomyza* sp. larvae on the transgenic and non-transgenic plants was not significantly different (Figure 7C). Also, both transgenic and non-transgenic plants were similarly damaged by the leafminer larvae, with no difference on the level of damage they caused on the plants (Figure 7D).

## Discussion

Common bean is a staple food in Brazil, with social and economic importance. The crop is produced all over the country, in three growing seasons per year, which means that insect pests have a favorable environment to reproduce and keep high populations throughout the year. Whitefly management in the common bean crop is particularly relevant, because this insect is a vector of viruses that can severely impair crop yield and grain quality (Souza et al., 2016). Chemical control of the insect population has been the most used control method, although alternatives have been developed, such as biological control and virus resistant common bean cultivars (Faria et al., 2016; Souza et al., 2018; Sani et al., 2020; Silva et al., 2022). Common bean cultivars with tolerance to the whitefly through antixenosis have been reported, resulting in reduced number of eggs, nymphs and adults sitting on the plants, in field assays (Silva et al., 2014, 2019; Hoshino et al., 2017; Jesus et al., 2021). In spite of the identification of these sources of tolerance in the common bean germplasm, no common bean cultivar has been developed or registered for resistance to the whitefly, to date. Using RNAi to silencing genes in the insect might be a more specific and durable strategy, with potential to cause high insect mortality. The whitefly *B. tabac*i is a highly efficient vector of plant viruses, able to transmit numerous viruses from mixed-infected plants with different levels of efficiency. Some of these viruses are transmitted by the whitefly in a non-persistently manner, which means that the virus acquisition period is very short (1 to 3 min). Insect probing behavior plays a major part in this mode of transmission, meaning that even brief probes can be sufficient for a quick plant-to-plant spread of these viruses. Considering this insect-virus mode of interaction, a strategy aiming to cause insect mortality is more promising for virus disease management. Plant genetic transformation for gene silencing *via* RNAi is a viable option to achieve significant insect mortality rates and it has been successfully used to obtain stable transgenic whitefly-resistant plants, such as tomato and lettuce (Ibrahim et al., 2017; Pizetta et al., 2021; Xia et al., 2021), but not common beans, so far.

Our results show the development of the first common bean transgenic line with tolerance to the whitefly *B. tabaci*. Stable transformation of grain legumes has been considered a challenge (De Clercq et al., 2002). Because the common bean *P. vulgaris* is recalcitrant to *in vitro de novo* regeneration from callus, *Agrobacterium tumefaciens-*mediated genetic transformation of common beans is still difficult to achieve and therefore, the most reliable technique that made it possible obtaining a commercial cultivar of transgenic common bean, to date, was particle bombardment (Aragão et al., 1996; Faria et al., 2016). This technique presents a lower transformation rate, compared to *A. tumefaciens-*mediated genetic transformation of other plant species (De Clercq et al., 2002). Accordingly, in our experiments, only two stable transgenic common bean lines were obtained, that is, which passed the transgene to their progeny, representing an efficiency rate of 0.02%. From those, only one of them presented a significant amount of targeting siRNA, associated with a significant insect mortality and silencing of the target gene in the insects. These results are in agreement with the gene silencing ability of the other transgenic plant species, lettuce and tomato, that our team previously engineered using the same genetic construction (Ibrahim et al., 2017; Pizetta et al., 2021). In lettuce, silencing of the whitefly *vATPase* gene was associated with higher mortality, from 83.8–98.1% (Ibrahim et al., 2017), while in tomato, insect mortality was similar to our current results with the common bean line Bt-22.5, about 60% (Pizetta et al., 2021). Silencing of the *vATPase* gene has been reported as an efficient method to interfere with survival and development of the whitefly *B. tabaci* (Upadhyay et al., 2011; Thakur et al., 2014), although it seems to be more effective on adult insects. Significant mortality of 2nd instar nymphs was reported for the transgenic tomato, while the transgenic lettuce also delayed the whitefly development from nymphs to pupae (Ibrahim et al., 2017; Pizetta et al., 2021). In the current work, no significant difference in the survival or development of the whitefly young stages was observed on the transgenic common bean lines (data not shown). The other common bean line obtained in the current work, named 11.5, although positive for the transgene, produced a smaller amount of siRNA and did not cause significant insect mortality. This might be related to the DNA integration site, number of transgene copies and other inherent obstacles of plant genetic transformation. The transgenic plants did not present any other phenotypical difference from the non-transgenic plants.

Conducting mortality experiments with adult whiteflies is challenging because *B. tabaci* is a small, fragile and highly mobile insect, which makes it difficult to handle the insects without damaging their stylets, for example, when using an insect aspirator. Also, it is difficult to visualize dead insects on the plant or in the soil because the tiny whiteflies disintegrate very quickly. Therefore, we tested different methods for the mortality experiments, such as using detached leaves, to reduce the space in which the dead insects would be located. However, the detached leaf experiments resulted in high mortality rates, which could be artificial. To check that, we developed another methodology to conduct the experiments using whole plants and minimal insect handling. For that, we exposed the plants to adult whiteflies for oviposition and then, we removed the adults and waited until the nymphs developed into a new generation of adults on the plants. This methodology has the advantage of minimally disturbing the insects while they develop from egg to adult on the transgenic plant, thus increasing the exposition time of insects to the transgene and also reducing the mortality due to random effects in the controls. Our results show insect mortality on the common bean line Bt-22.5 was higher on the experiment with detached leaves, in comparison with the experiment with whole plants. Even so, the mortality in the whole plant indicates a good level of tolerance to the insect, which can contribute to pest management, along with other management tools already available. Accordingly, in other studies the two methods of bioassays generally show a positive data correlation, although in some cases the responses point to more or less pronounced effects, depending on the target organism (Sharma et al., 2005; Michel et al., 2010; Miller-Butler et al., 2018). Also, for distinguishing resistant from susceptible genotypes, the two methodologies generally correlate well. This is in agreement with our observations, which show that the experiments with detached leaves were useful to select the most promising resistant lines for further confirmation with the whole plant methodology. Some variation can still be found in future experiments, depending on uncontrolled field and climate conditions.

In the preference assay, the number of adult insects and the number of eggs were reduced on the transgenic common bean line Bt-22.5, which is in agreement to the lower oviposition reported for the transgenic lettuce and tomato, genetically engineered with the same RNAi construct (Ibrahim et al., 2017; Pizetta et al., 2021). Corroborating this result, in the experiment to look at the whitefly generations during the cycle of the common bean plants, the total number of adults and the number of adults per sampling date were lower on the transgenic plants, suggesting that these plants contributed to reduce the whitefly population and the start of the second generation. A similar pattern was observed in the study with the transgenic lettuce resistant to the whitefly, although in that study, the number of insects from all stages were lower on the transgenic plants (Ibrahim et al., 2017). However, most studies on the development of transgenic plants in the literature do not report the effect of gene silencing over insect generations, or during the plant life cycle.

Regarding virus transmission, our results show that feeding on the transgenic plant did not affect the whitefly ability to transmit two viruses, in different modes (circulative and non-circulative). In fact, silencing the *vATPase* gene in the insect was not expected to affect virus transmission, because this gene has not been reported to be as relevant for the vectoring ability of Hemipterans as other genes, such as *HSP70*, *cathepsin B*, *cyclophilin B* and *α-glucosidase* (Götz et al., 2012; Chen et al., 2016; Pinheiro et al., 2017; Hasegawa et al., 2018; Kanakala et al., 2019; Lu et al., 2021). A transcriptomic study showed that the *vATPase* gene was not differentially expressed in whiteflies that acquired the tomato chlorosis virus (ToCV), compared with insects that fed on non-infected plants (Kaur et al., 2017). The variation in the level of virus symptoms in the plants that we observed in the present study are similar to natural infections in the field and may be explained by other factors, for example, environmental effects, number of insects feeding on each plant and viral load variation among insects.

Furthermore, the transgenic common bean line Bt-22.5 did not cause unexpected effects on the reproduction and development of three non-target organisms. Two of these insect species are also considered as insect pests: the black aphid *A. craccivora*, which has a feeding habit similar to the whitefly, and the leafminer *Liriomyza* sp. Our results are in agreement with the non-target assays conducted for the whitefly-resistant transgenic tomato lines with different non-target organisms, also insect pests, such as the green peach aphid *Myzus persicae*, the spider mite *Tetranychus urticae* and the tomato leafminer *Tuta absoluta* (Pizetta et al., 2021; Xia et al., 2021). This suggests that even for the organisms with similar *vATPase* gene sequences, silencing of the *B. tabaci vATPase* was specific to the target species. The other non-target organism evaluated is a beneficial insect, the parasitoid of whitefly nymphs *E. formosa*. Our results show that silencing the whitefly *vATPase* did not affect the ability of the parasitoid to reproduce in the whitefly nymphs.

In summary, our results show that the transgenic common bean line Bt-22.5 can contribute with the management of the whitefly, along with other management tools, with potential to reduce the need of numerous insecticide sprays. The next step will be crossing the line Bt-22.5 with elite genotypes from the Embrapa common bean breeding program, to introduce the whitefly tolerance into common bean genotypes along with other desirable agronomic traits, such as high yield, grain quality and multiple virus resistance (BGMV, BCMV and CPMMV), as it was recently reported (Silva et al., 2022).

## Conclusion

Our results show the development of a stable transgenic common bean plant tolerant to the whitefly *B. tabaci* that can eventually be used as an additional management tool in Integrated Pest Management (IPM). Plant-mediated silencing of the *B. tabaci vATPase* gene conferred a reasonable level of whitefly-tolerance to the transgenic common bean line. The transgenic plants did not show any other phenotypical difference, nor negative effects on the evaluated non-target insect species. This transgenic common bean event represents a sustainable pest management strategy that might contribute to avoid the intensive use of insecticides and to reduce environmental and financial costs.

## Data availability statement

The datasets generated for this study can be found in the Embrapa’s research data repository, SIEXP [https://www.siexp.cnptia.embrapa.br/siexp-mweb/]. Transgenic seeds will be made available after the development and release of a new commercial cultivar.

## Author contributions

PP, JF, FA, AF, JB, LH, and TS contributed to the conception and design of the study. JF, AF, GC, MM, FA, PP, and EF conducted the genetic transformation assays and *in vitro* regeneration of transgenic plants. AF, AZ, JB, PP, and JS contributed to the conduction of the bioassays with insects. AF, FA, CP, and PP conducted the siRNA quantification assays. JS, AF, and PP performed the statistical analysis and elaborated graphs and figures. PP, FA, LH, and TS contributed with research grant funding application and management. AF wrote the first draft of the manuscript. PP wrote the final version of the manuscript. All authors contributed to the article and approved the submitted version.

## Funding

This work was supported by Brazilian Agricultural Research Corporation – Embrapa (Grant nos. 20.18.03.035.00.00, 10.19.00.089.00.00.00, and 20.18.04.008.00.00). AF, MM, AZ, TS and FA are supported by the CNPq, the Brazilian Council for Scientific and Technological Development. The funders were not involved in the study design, collection, analysis, interpretation of data, the writing of this article or the decision to submit it for publication.

## Conflict of interest

The authors declare that the research was conducted in the absence of any commercial or financial relationships that could be construed as a potential conflict of interest.

## Publisher’s note

All claims expressed in this article are solely those of the authors and do not necessarily represent those of their affiliated organizations, or those of the publisher, the editors and the reviewers. Any product that may be evaluated in this article, or claim that may be made by its manufacturer, is not guaranteed or endorsed by the publisher.

## Supplementary material

Supplementary Figure 1 Plasmid map displaying the organization of the whitefly *Bemisia tabaci vATPase* partial gene sequence cloned in sense and antisense, intercalated by intron 3 of the *malate synthase* gene of *Arabidopsis thaliana* for the production of siRNAs and the Atahas gene, with the complete promoter and terminator of *A. thaliana*, for the selection of transgenic plants, conferring resistance to the herbicide imazapyr.

Supplementary Table S1 Agronomic traits of the transgenic common bean line 22.5 and the non-transgenic control line.
