# Supplementary figures and images for: Whitefly-tolerant transgenic common bean (Phaseolus vulgaris) line
Source: Front Plant Sci. 2022 Aug 25;13:984804. doi: 10.3389/fpls.2022.984804 (PMC9453422; doi:10.3389/fpls.2022.984804)

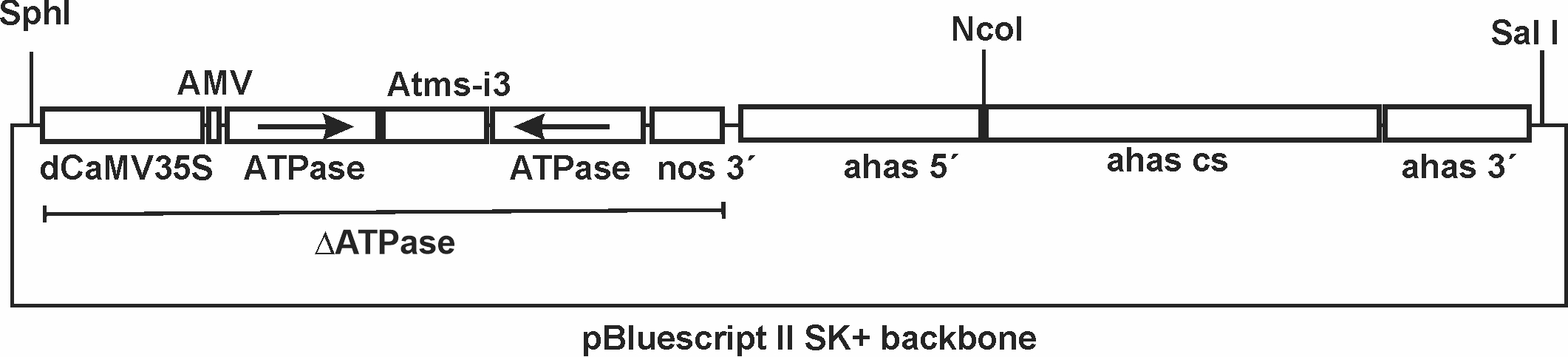

Supplement: Supplementary Figure 1 — Plasmid map displaying the organization of the whitefly Bemisia tabaci vATPase partial gene sequence cloned in sense and antisense, intercalated by intron 3 of the malate synthase gene of Arabidopsis thaliana for the production of siRNAs and the Atahas gene, with the complete promoter and terminator of A. thaliana, for the selection of transgenic plants, conferring resistance to the herbicide imazapyr. [file Image_1.JPEG]
