# Supplementary material for: Whitefly-tolerant transgenic common bean (Phaseolus vulgaris) line
Source: Front Plant Sci. 2022 Aug 25;13:984804. doi: 10.3389/fpls.2022.984804 (PMC9453422; doi:10.3389/fpls.2022.984804)
Supplement: Supplementary Table S1 — Agronomic traits of the transgenic common bean line 22.5 and the non-transgenic control line. [file Table_1.DOCX]

Table S1. Agronomic traits of the transgenic common bean line 22.5 and the non-transgenic control line.

| Agronomic trait | Transgenic line | Non-transgenic line | p-value |
| --- | --- | --- | --- |
| Number of pods/plant (average ± SE) | 3.89 ± 0.51 | 3.28 ± 0.36 | 0.3589 |
| Average number of seeds/pod (average ± SE) | 2.17 ± 0.13 | 2.12 ± 0.14 | 0.8109 |
| Mass of 100 seeds (g) (average ± SE) | 12.72 ± 1.03 | 13.64 ± 0.55 | 0.5546 |
